# A novel αB-crystallin R123W variant drives hypertrophic cardiomyopathy by promoting maladaptive calcium-dependent signal transduction

**DOI:** 10.3389/fcvm.2023.1223244

**Published:** 2023-06-26

**Authors:** Chun Chou, Gregory L. Martin, Gayani Perera, Junya Awata, Amy Larson, Robert Blanton, Michael T. Chin

**Affiliations:** ^1^ Department of Medicine, Tufts University School of Medicine, Boston, MA, United States; ^2^ Molecular Cardiology Research Institute, Tufts Medical Center, Boston, MA, United States

**Keywords:** hypertrophic cardiomyopathy, cardiac hypertrophy, cryab, calcineurin, NFAT, transverse aortic constriction, cardiac fibrosis

## Abstract

Hypertrophic cardiomyopathy (HCM) is the most common inherited cardiovascular disorder affecting 1 in 500 people in the general population. Characterized by asymmetric left ventricular hypertrophy, cardiomyocyte disarray and cardiac fibrosis, HCM is a highly complex disease with heterogenous clinical presentation, onset and complication. While mutations in sarcomere genes can account for a substantial proportion of familial cases of HCM, 40%–50% of HCM patients do not carry such sarcomere variants and the causal mutations for their diseases remain elusive. Recently, we identified a novel variant of the alpha-crystallin B chain (*CRYAB*^R123W^) in a pair of monozygotic twins who developed concordant HCM phenotypes that manifested over a nearly identical time course. Yet, how *CRYAB*^R123W^ promotes the HCM phenotype remains unclear. Here, we generated mice carrying the *Cryab*^R123W^ knock-in allele and demonstrated that hearts from these animals exhibit increased maximal elastance at young age but reduced diastolic function with aging. Upon transverse aortic constriction, mice carrying the *Cryab*^R123W^ allele developed pathogenic left ventricular hypertrophy with substantial cardiac fibrosis and progressively decreased ejection fraction. Crossing of mice with a *Mybpc3* frame-shift model of HCM did not potentiate pathological hypertrophy in compound heterozygotes, indicating that the pathological mechanisms in the *Cryab*^R123W^ model are independent of the sarcomere. In contrast to another well-characterized *CRYAB* variant (R120G) which induced Desmin aggregation, no evidence of protein aggregation was observed in hearts expressing *CRYAB*^R123W^ despite its potent effect on driving cellular hypertrophy. Mechanistically, we uncovered an unexpected protein-protein interaction between CRYAB and calcineurin. Whereas CRYAB suppresses maladaptive calcium signaling in response to pressure-overload, the R123W mutation abolished this effect and instead drove pathologic NFAT activation. Thus, our data establish the *Cryab*^R123W^ allele as a novel genetic model of HCM and unveiled additional sarcomere-independent mechanisms of cardiac pathological hypertrophy.

## Introduction

Hypertrophic cardiomyopathy (HCM), characterized by myocyte hypertrophy resulting in the thickening of the ventricular wall, decreased ventricular volume and diastolic dysfunction, has been recognized as the most common inherited cardiovascular disease, affecting 1 in every 500 young individuals (1). Inheritance is archetypally considered autosomal dominant with high penetrance in 50%–60% of patients (2, 3). The relatively high concordance has enabled multiple genome wide association studies to identify causal genetic mutations that underly HCM pathogenesis in large and unrelated cardiomyopathy families (4–8). Among the chromosomal loci that have been linked to HCM, the majority of mutations occur in genes encoding cardiac sarcomere proteins (8), including β-myosin heavy chain, cardiac myosin binding protein C and cardiac troponin T, which have well described roles in cardiomyocyte excitation-contraction coupling (9). While extensive studies have been conducted to elucidate the molecular mechanisms by which sarcomere protein variants cause HCM, less than 30% of patients with established diagnosis of HCM carry sarcomere gene mutations classified as or presumed to be pathogenic (10). In fact, isolated and sporadic HCM cases in which the proband does not carry any known HCM mutations may account for up to 40% of all HCM cases (11). Thus, the sarcomere-centric paradigm of HCM pathogenesis does not fully encapsulate the pathogenic mechanisms of HCM and many causal mutations for HCM still remain elusive (12).

Decades ago, a genetic linkage study identified a novel variant of the alpha-crystallin B chain (CRYAB) as a cause of HCM in a French family with myopathy in multiple organs (13). CRYAB is a member of the small heat shock binding protein (sHSBP) family and serves as a molecular chaperone with a wide spectrum of biological functions in cardiomyocytes, ranging from modulating calcium signaling (14), preventing protein aggregation (15–17), regulating autophagy (18, 19), to maintaining cellular survival (20). Intriguingly, under physiologic conditions, CRYAB in fact spontaneously organize into dimers and oligomers with little chaperone activity (21, 22). Stress signals such as elevated temperature disrupt the oligomeric complex, thereby exposing the unstructured N- and C-terminal domain, which provide the necessary chaperone function (23, 24). It thus appears that maintenance of CRYAB dimer/oligomer state under physiologic conditions may be essential in preventing inappropriate interaction with other proteins. Stabilization of CRYAB dimers is critically dependent on salt-bridges formed by arginine at position 120 and aspartic acid at position 109 on respective dimerization partners (21, 22) with non-synonymous mutations of R120 and D109 causally linked to various familial cardiomyopathies (25). In particular, substitution of R120 for glycine (hereon designated as *CRYAB*^R120G^) was identified as a causal mutation in Desmin related myopathy characterized by intracellular accumulation of spheroid inclusion bodies consisted of Desmin (26). Consistently, CRYAB^R120G^ expression was sufficient to induce Desmin aggregation in murine cardiomyocytes and mice over-expressing CRYAB^R120G^ specifically in cardiomyocytes developed spontaneous cardiac dysfunction and succumbed to disease at 32 weeks of age (27). Cryoelectron microscopy revealed abnormal quaternary structure of CRYAB^R120G^ with an apparent molecular weight more than doubled compared to wild-type CRYAB, suggesting R120G mutation disrupts proper oligomerization of CRYAB (28). Another variant of CRYAB where D109 was mutated to glycine (hereon designated as *CRYAB*^D109G^) has been linked to familial restrictive cardiopathy (29), with a presumed mechanism of dimer/oligomer destabilization considering the critical ionic interaction between D109 and R120. Collectively, these seminal works identified *CRYAB* as a potential hotspot for cardiomyopathy-inducing gene mutations. Recently, we reported a case of monozygotic twins who developed concordant HCM phenotypes that manifested over a nearly identical time course (30). Among the non-synonymous variants of HCM-associated genes, we identified yet another variant of CRYAB (OMIM 123590) where the arginine at amino acid 123 was substituted for tryptophan (hereon designated as *CRYAB*^R123W^). While previously classified as a variant of unknown significance, the high concordance of diease manifestation by the pair of monozygotic twins strongly implies a causal role of *CRYAB*^R123W^ in HCM pathogenesis.

In this study, we demonstrated that CRYAB^R123W^ readily promoted cellular hypertrophy *in vitro* and mice carrying the *Cryab*^R123W^ allele spontaneously developed diastolic dysfunction with aging. Although hearts from these animals did not undergo hypertrophy at steady-state, pressure-overload by transaortic constriction (TAC) markedly induced cardiac hypertrophy and parenchymal fibrosis, associated with progressive systolic dysfunction. Mechanistically, CRYAB binds to calcineurin in co-immunoprecipitation assays, and such interaction may be critical for CRYAB-dependent suppression of maladaptive calcium signaling in response to pressure-overload. Intriguingly, the R123W mutation abolished such cardioprotective effects and instead converted CRYAB into an activator of pathologic calcium signaling.

## Results

### CRYAB^R123W^ enhances contractility early but impairs diastolic function with aging

**Figure 1:**
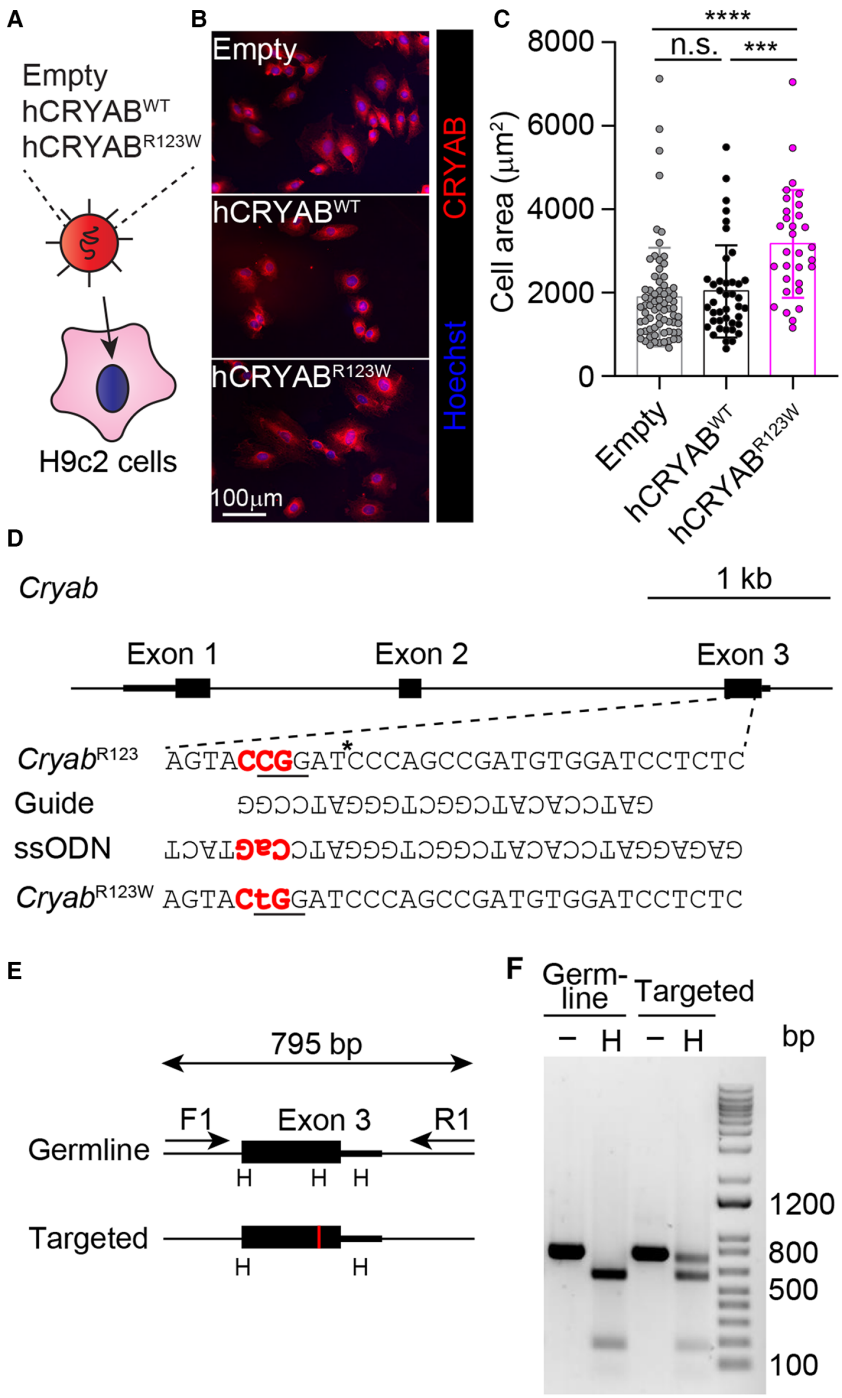
Generation of the *Cryab*^R123W^ knock-in allele. (**A**) Transduction of H9c2 cells with lentiviruses expressing the indicated constructs. (**B**) Immunofluorescence microscopic images showing CRYAB expression in H9c2 cardiomyofibroblasts transduced with empty, human wild-type CRYAB (hCRYAB^WT^)- or R123W variant (hCRYAB^R123W^)-expressing lentivirus. Data are representative of two independent experiments. (**C**) Cross-section areas of H9c2 cells transduced with indicated lentiviruses. Data are pooled from two independent experiments. (**D**) A schematic diagram showing the generation of the *Cryab*^R123W^ knock-in allele via CRISPR/Cas9-mediated homology directed repair. The trinucleotides in red denote the protospacer adjacent motif (PAM). The asterisk indicates the cleavage site by the Cas9 nuclease. The underlined trinucleotides encode the target amino acid and the desired missense mutation. (**E**) A screening strategy to identify targeted founders. PCR products of targeted allele differed from germ line configuration by a loss of HpaI (**H**) restriction enzyme site, denoted by the red vertical line. (**F**) Restriction fragment length polymorphisms analysis of germline and targeted animals. PCR fragments spanning the exon of interest before and after HpaI digestion were analyzed by agarose gel electrophoresis. Data are mean ± s.d. Kruskal-Wallis test (**C**). ***P < 0.001; ****P < 0.0001.

**Figure 2:**
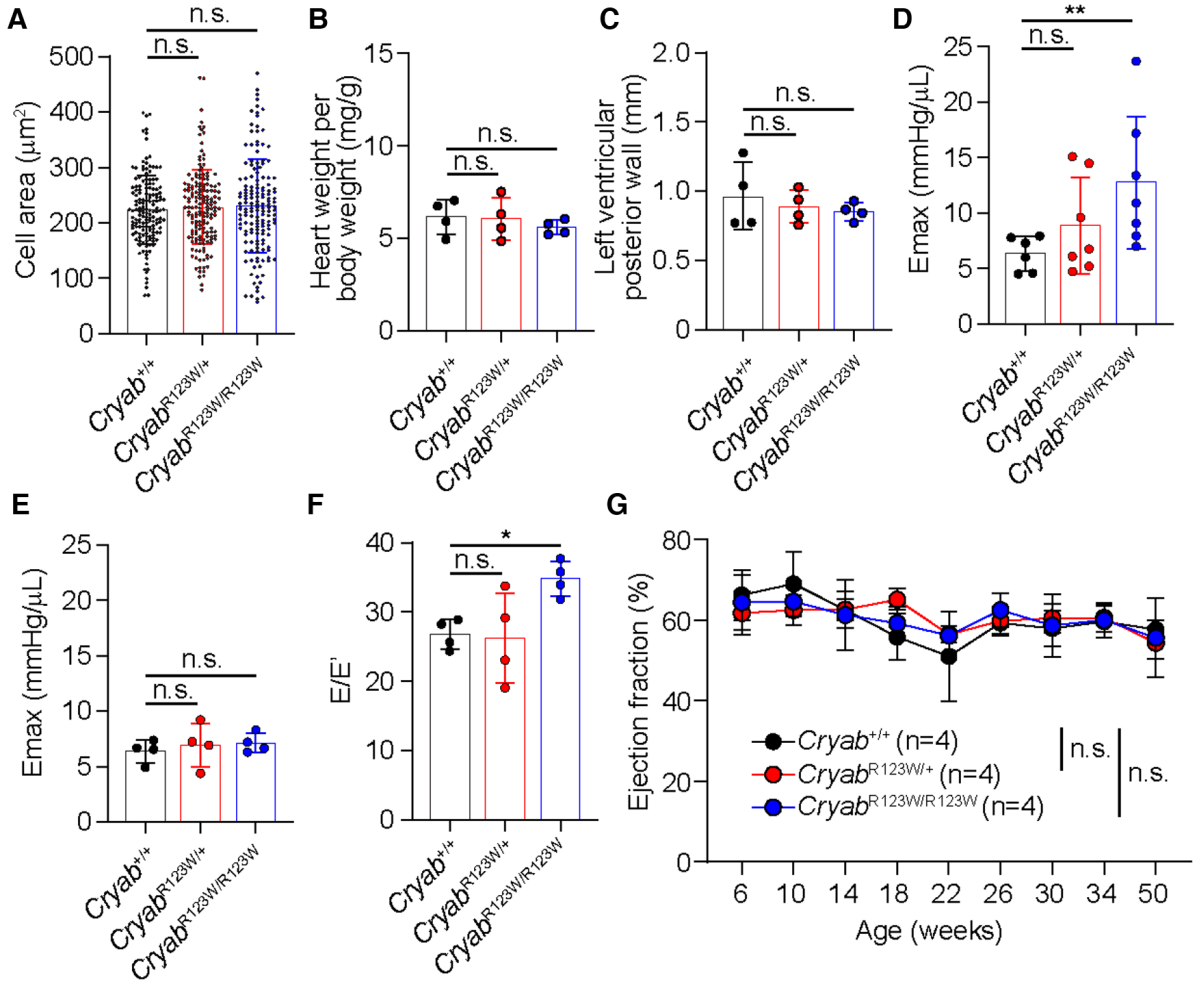
CRYAB^R123W^-expressing mice spontaneously developed diastolic dysfunction. (**A**) Cross section areas of cardiomyocytes from *Cryab*^+/+^, *Cryab*^R123W/+^ and *Cryab*^R123W/R123W^ mice between 8- to 12- weeks of age. Data are pooled from two independent experiments. (**B**) Normalized heart weight by body weight in *Cryab*^+/+^, *Cryab*^R123W/+^ or *Cryab*^R123W/R123W^ mice at 8- to 12-weeks of age. Data are pooled from two independent experiments. (**C**) Left ventricular posterior wall thickness measured by echocardiogram in mice of indicated genotypes at 8- to 12-weeks of age. Data are pooled from two independent experiments. (**D,E**) Left ventricular maximal elastance (Emax) of hearts from *Cryab*^+/+^, *Cryab*^R123W/+^ or *Cryab*^R123W/R123W^ mice at 8- to 10-weeks (**D**) or 50- to 52-weeks (**E**) of age derived from volume-pressure loop data during the pre-load reduction stage. Data are pooled from three independent experiments. (**F**) E/E′ of hearts from indicated genotypes at 25- to 30-weeks of age derived from transmural inflow Doppler indexes. Data are pooled from two independent experiments. (**G**) Longitudinal analysis of left ventricular ejection fraction of *Cryab*^+/+^, *Cryab*^R123W/+^ and *Cryab*^R123W/R123W^ hearts. Data are pooled from two independent experiments. Data are mean ± s.d. Kruskal-Wallis test (**A–F**). Two-way ANOVA test (**G**). *P < 0.05; n.s., not significant.

To test whether the CRYAB^R123W^ variant is sufficient to drive cardiomyocyte hypertrophy, H9c2 cells derived from rat cardiomyofibroblasts were engineered to express human wild-type CRYAB (hCRYAB^WT^) or the R123W variant (hCRYAB^R123W^) via lentiviral transduction (Figure 1A). Consistently, ectopic expression of hCRYAB^R123W^ variant but not hCRYAB^WT^ resulted in substantial cellular hypertrophy (Figures 1B,C). To evaluate whether CRYAB^R123W^ drives HCM pathogenesis *in vivo*, we generated knock-in mice carrying the *Cryab*^R123W^ mutation using CRISPR/Cas9-mediated homology directed repair (Figures 1D–F). While no cardiomyocyte or cardiac hypertrophy was observed in heterozygous or homozygous mice at steady-state (Figures 2A–C), Emax was already increased in *Cryab*^R123W/R123W^ mice and to a lesser extent in *Cryab*^R123W/+^ counterparts at eight weeks of age (Figure 2D). Intriguingly, enhanced Emax was no longer observed in aged mice carrying the *Cryab*^R123W^ allele (Figure 2E). Rather, *Cryab*^R123W/R123W^ mice showed an increased E/E′ (Figure 2F), indicating diastolic dysfunction which is commonly seen in almost all HCM patients (31). Together, these data established CRYAB^R123W^ as a potential disease-causing variant in human HCM with its pathogenic function likely conserved between human and rodents.

### CRYAB^R123W^ drives maladaptive cardiac remodeling upon pressure-overload

**Figure 3:**
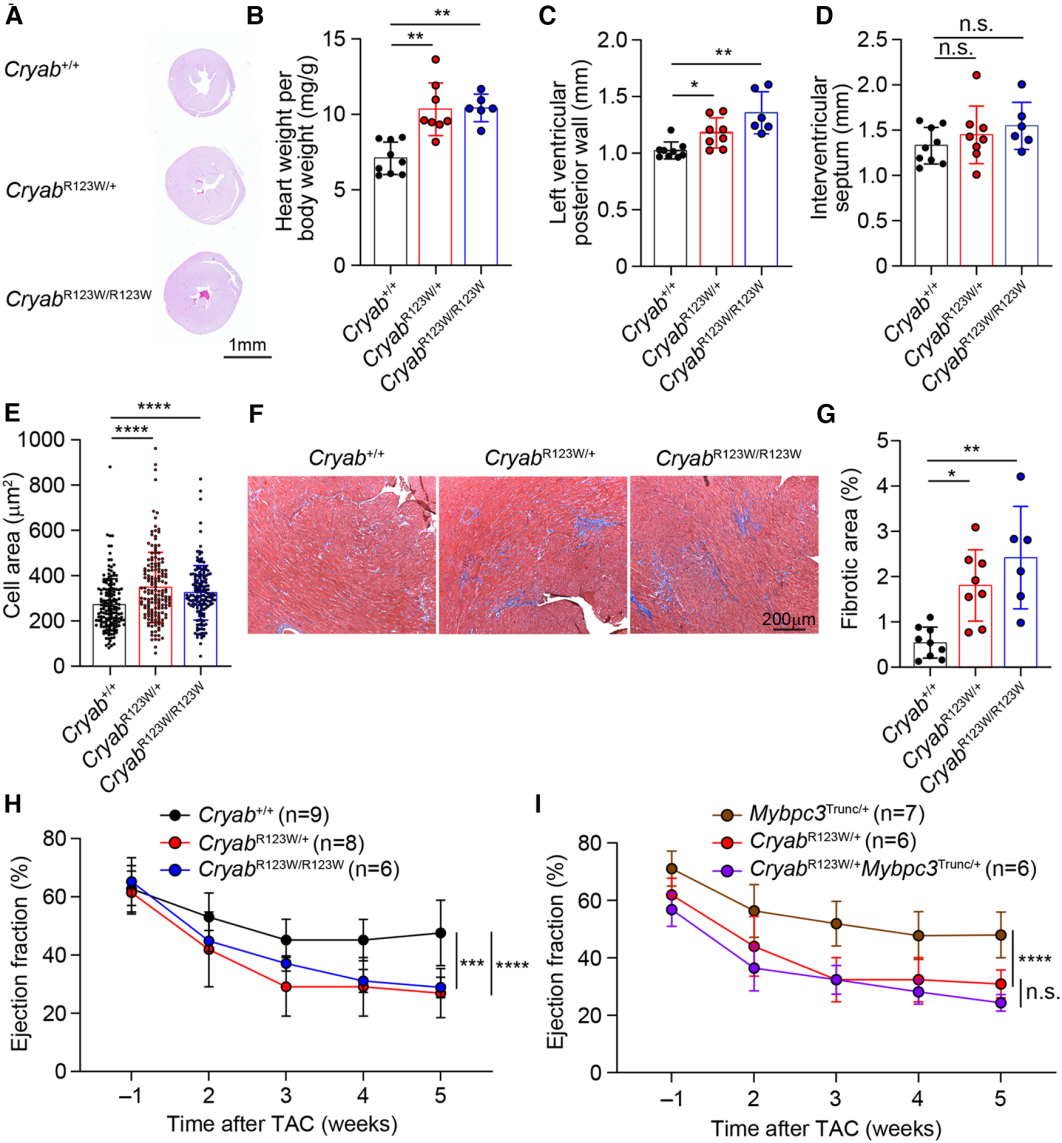
CRYAB^R123W^ drives maladaptive cardiac remodeling in response to pressure overload. (**A**) Hematoxylin/Eosin-stained cross-section of mid-papillary regions from *Cryab*^+/+^, *Cryab*^R123W/+^ or *Cryab*^R123W/R123W^ hearts five weeks after TAC. Data are representative of three independent experiments. (**B**) Normalized heart weight by body weight in mice of indicated genotypes five weeks after TAC. Data are pooled from three independent experiments. (**C,D**) Statistical analysis of left ventricular posterior wall thickness and interventricular septum thickness measured by echocardiogram in mice of indicated genotypes five weeks after TAC. Data are pooled from three independent experiments. (**E**) Cross section areas of cardiomyocytes from mice of indicated genotypes five weeks after TAC. Data are pooled from three independent experiments. (**F,G**) Masson trichome stained histological sections and percentage of fibrotic areas in hearts from indicated genotypes five weeks after TAC. Data are representative (**F**) and pooled (**G**) from three independent experiments. (**H**) Statistical analysis of left ventricular ejection fraction of *Cryab*^+/+^, *Cryab*^R123W/+^ and *Cryab*^R123W/R123W^ hearts one week prior to and weekly after TAC. Data are pooled from three independent experiments. (**I**) Left ventricular ejection fraction of *Mybpc3*^Trunc/+^, *Cryab*^R123W/+^ and *Cryab*^R123W/+^*Mybpc3*^Trunc/+^ hearts at indicated time prior to or after TAC. Data are pooled from three independent experiments. Data are mean ± s.d. Kruskal-Wallis test (**B–E,G**). Two-way ANOVA test (**H,I**). *P < 0.05; **P < 0.01; ***P < 0.001; ****P < 0.0001; n.s., not significant.

While the monozygotic twins carrying the heterozygous *CRYAB^R123W^* mutation developed clinically significant HCM at a young age, no histologic or echocardiographic evidence of significant HCM was observed throughout the lifespan of mice harboring the *Cryab*^R123W^ allele at steady state (Figure 2G and data not shown). Notably, only a few genetic mouse models of HCM to date exhibited spontaneous cardiac hypertrophy and almost all were driven by mutated sarcomere genes expressed from a transgene rather than the endogenous loci (32–35). Knock-in mice for a truncated form of *Mybpc3*, one of the most commonly mutated disease-causing genes of HCM, in fact did not develop spontaneous cardiac hypertrophy. Rather, the hypertrophic phenotype was only evident upon pressure-overload by TAC (36). Indeed, both *Cryab*^R123W/+^ and *Cryab*^R123W/R123W^ mice underwent marked cardiac hypertrophy after TAC compared to wild-type controls (Figures 3A,B). Of note, the hypertrophic phenotype appears to be circumferential as often observed in models of pressure overload (Figures 3A,C). Asymmetric hypertrophy, which preferentially affects the interventricular septum in HCM patients is rarely reproduced in mouse models of HCM and was expectedly not observed in the *Cryab*^R123W^ model (Figure 3D). Microscopically, cardiomyocytes from mice carrying the *Cryab*^R123W^ allele underwent greater extent of cellular hypertrophy compared to wild-type counterparts (Figure 3E). Furthermore, hearts from *Cryab*^R123W/+^ and *Cryab*^R123W/R123W^ mice demonstrated larger areas of parenchymal fibrosis compared to control animals (Figures 3F,G). Thus, the *Cryab*^R123W^ mouse model recapitulated key features of human HCM.

Systolic dysfunction has been reported in a small proportion of patients with HCM and is associated with poor prognosis (37, 38). Intriguingly, *Cryab*^R123W^ mice progressively developed systolic dysfunction after TAC (Figure 3H). In fact, the decline in ejection fraction was markedly more severe compared to that caused by two truncation variants of MYBPC3, identified by others (36) and our lab (Supplementary Figure S1 and Figure 3I). Importantly, systolic function was not further impaired in *Cryab*^R123W/+^*Mybpc*3^Trunc/+^ mice compared to *Cryab*^R123W/+^ counterparts (Figure 3I), together suggesting that CRYAB may promote HCM through sarcomere-independent mechanisms. Notably, while *Cryab*-deficient mice also exhibited pathologic cardiac hypertrophy with systolic dysfunction upon pressure overload (14), such phenotype was not seen in *Cryab*^null/+^ mice but readily evident in *Cryab*^R123W/+^ animals, indicating that CRYAB^R123W^ represents a pathologic rather than loss-of-function variant. These data thus demonstrated that the CRYAB^R123W^ variant actively promotes maladaptive cardiac remodeling in response to pressure-overload.

### CRYAB^R123W^ and CRYAB^R120G^ drive cardiomyopathy through distinct mechanisms

**Figure 4:**
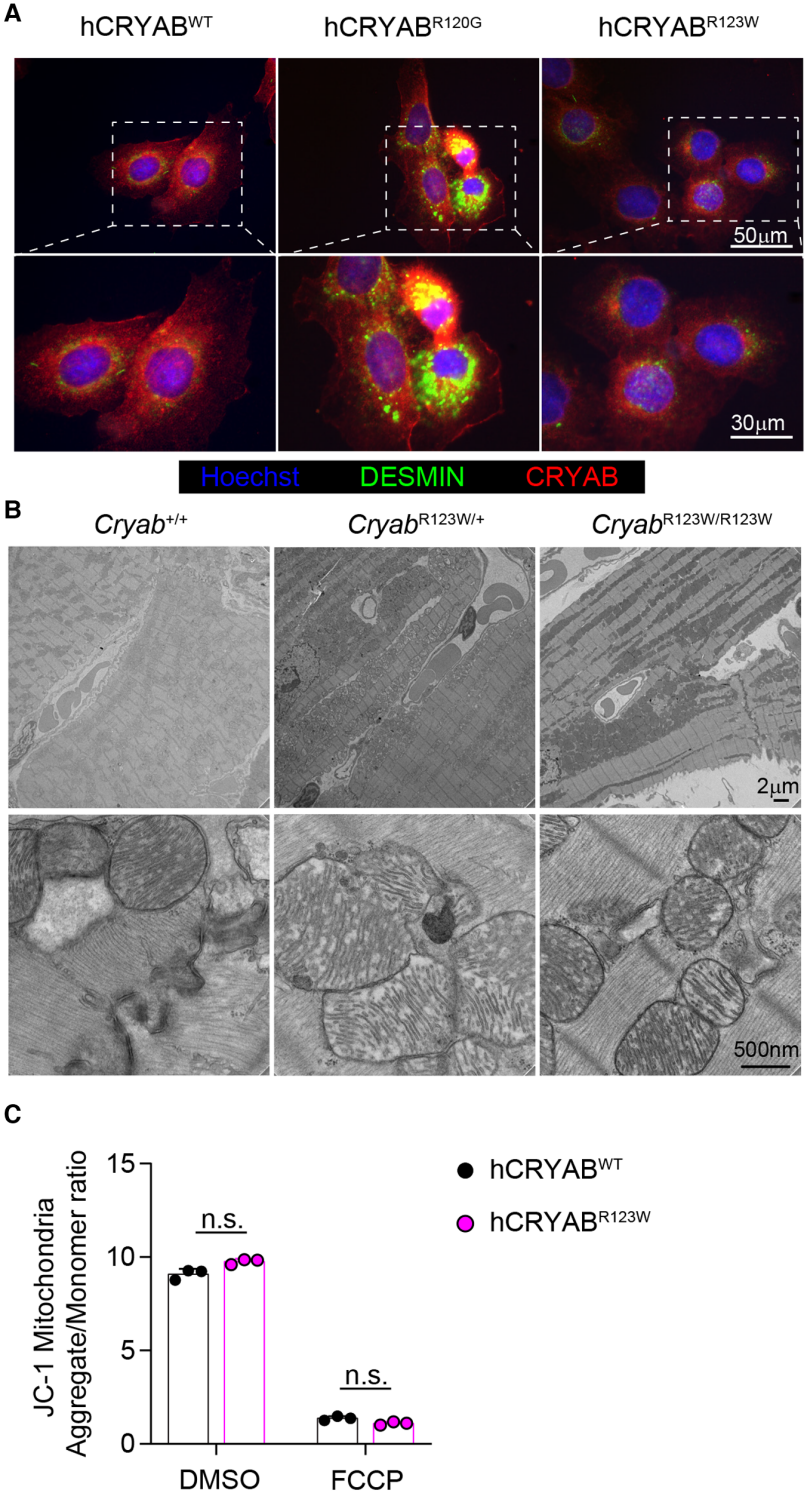
CRYAB^R123W^ does not induce protein aggregation. (**A**) Immunofluorescence microscopic images showing CRYAB and DESMIN expression in hCRYAB^WT^-, hCRYAB^R120G^-, and hCRYAB^R123W^-expressing H9c2 cells. Data are representative of two independent experiments. (**B**) Electron microscopy images of heart sections from *Cryab*^+/+^, *Cryab*^R123W/+^ or *Cryab*^R123W/R123W^ mice at six- to eight-weeks of age. (**C**) Statistical analysis of mitochondria aggregates to monomer ratio in hCRYAB^WT^- and hCRYAB^R123W^-expressing H9c2 cells treated with DMSO or an electron transport chain uncoupler FCCP. Data are pooled from two independent experiments. Data are mean ± s.d. Mann-Whitney test (**C**). n.s., not significant.

Another pathogenic CRYAB variant with substitution of amino acid arginine 120 for glycine (CRYAB^R120G^) has been linked to familial Desmin myopathy, characterized by intrasarcoplasmic accumulation of Desmin aggregates (13). However, distinct from *Cryab*^R123W^ mice which developed cardiac hypertrophy upon pressure-overload, mice expressing the CRYAB^R120G^ variant predominantly succumbed to spontaneously developed dilated cardiomyopathy at young age (27). Despite these disparate phenotypes, the close proximity of the two mutated amino acids nevertheless raises the possibility that CRYAB^R123W^ may also induce Desmin aggregation. Intriguingly, overexpression of hCRYAB^R123W^ did not result in perinuclear aggregation of Desmin, which was otherwise robustly induced by hCRYAB^R120G^ (Figure 4A) (39). In fact, unlike CRYAB^R120G^-expressing hearts (27), no protein aggregates were detected in heart tissues from *Cryab*^R123W/+^ or *Cryab*^R123W/R123W^ mice (Figure 4B). Additionally, while CRYAB^R120G^ has been shown to disrupt mitochondrial membrane potential (40, 41) such a defect was not observed in hCRYAB^R123W^-expressing cardiomyocytes (Figure 4C). Thus, CRYAB^R123W^ and CRYAB^R120G∫^ drive cardiomyopathy through distinct mechanisms.

### CRYAB interacts with calcineurin

**Figure 5:**
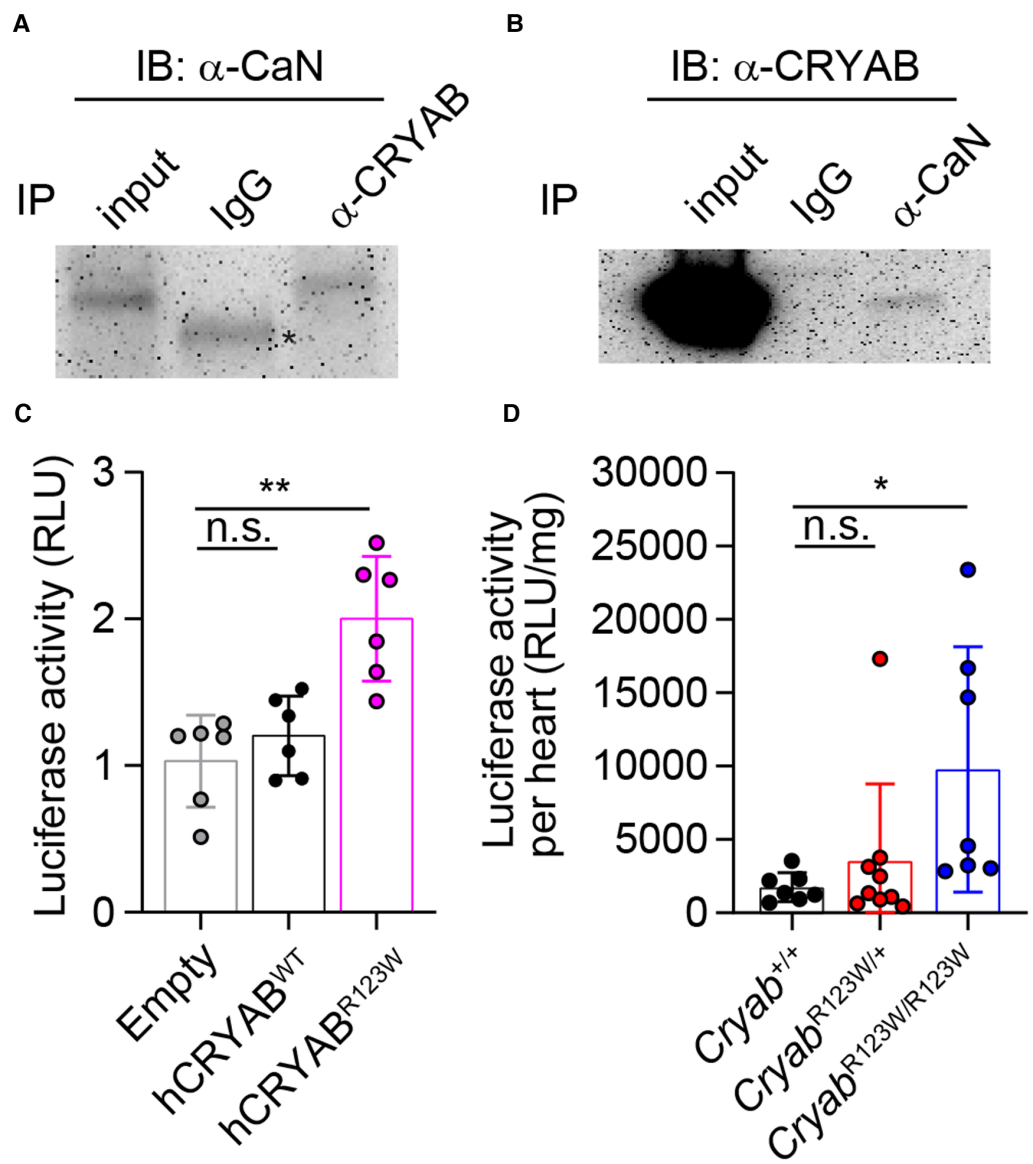
CRYAB^R123W^ promotes pathologic calcium-dependent signaling transduction. (**A**) Immunoblot analysis of anti-CRYAB immunoprecipitates (IP) and total cell lysate (input) from H9c2 cardiomyofibroblasts with an anti-Calcineurin (CaN) antibody and a peroxidase-conjugated anti-mouse IgG light chain-specific secondary antibody. Data is representative of three independent experiments. (**B**) Anti-CaN IP and input from H9c2 cardiomyofibroblasts were analyzed by immunoblot assay using an anti-CRYAB antibody and a peroxidase-conjugated anti-mouse IgG Fc-specific secondary antibody. Data are representative of two independent experiments. (**C**) Statistical analysis of NFAT-luciferase activity in empty, hCRYAB^WT^- or hCRYAB^R123W^-expressing H9c2 cells one day after co-transfection with a NFAT-inducible firefly luciferase expression plasmid and a constitutive expression plasmid encoding renilla luciferase. The relative luciferase unit is calculated as a ratio between firefly and renilla luciferase activity. Data are pooled from three independent experiments. (**D**) Normalized firefly luciferase activity by heart weight in steady state six- to eight-week-old *Cryab*^+/+^, *Cryab*^R123W/+^ or *Cryab*^R123W/R123W^ mice carrying a transgene in which firefly luciferase expression is under the control of myosin heavy chain promoter and tandem NFAT enhancer elements. Data are pooled from three independent experiments. Data are mean ± s.d. Kruskal-Wallis test (**C,D**). *P < 0.05; **P < 0.01; n.s., not significant.

Myriad molecular mechanisms have been implicated in HCM pathogenesis (42). In particular, enhanced calcium-dependent signaling was consistently observed in myectomized heart tissues from HCM patients regardless of whether sarcomere mutations are present (43, 44). Similarly, an increase in calcium-dependent signaling was also observed in TAC-induced pathologic cardiac hypertrophy in mice (45). Interestingly, *Cryab* expression was induced by pressure overload and its upregulation appears to be a cardio-protective adaptation that mitigates an otherwise pathologic hyperactivation of calcium signaling and the ensuing cellular hypertrophy (14). These findings thus ascribe *CRYAB* an anti-hypertrophy role in part through curbing maladaptive calcium-dependent signaling. Mechanistically, CRYAB inhibited nuclear translocation of the transcription factor NFAT (45). As one of the most abundantly expressed chaperone proteins in cardiomyocytes (46), CRYAB has been shown to interact with a wide variety of proteins (47). Yet, besides reported interactions with components of cytoskeleton (48) and effectors of apoptosis (49), whether CRYAB interacts with mediators of the calcium-dependent signaling cascade remains unclear. To this end, we performed a targeted survey of the CRYAB protein interactome focusing on key regulators of the calcium-dependent signaling cascade. Interestingly, immunoprecipitation of CRYAB revealed a robust interaction with calcineurin, but not calmodulin or NFAT (Figure 5A and data not shown). Reciprocally, CRYAB was also detected in a calcineurin-containing protein complex (Figure 5B). Thus, these findings suggest that CRYAB may dampen pathologic calcium-dependent signaling via modulation of calcineurin activity.

### CRYAB^R123W^ enhances calcium-dependent signaling

To investigate whether the R123W mutation alters CRYAB's ability to suppress calcium-dependent signal transduction, control, hCRYAB^WT^- or hCRYAB^R123W^-expressing H9c2 cardiomyofibroblasts were transfected with a NFAT-luciferase reporter plasmid in which firefly luciferase expression is under the control of a myosin heavy chain promoter and tandem calcium-responsive enhancer elements (45). Strikingly, the hCRYAB^R123W^ variant readily induced calcium-dependent signaling even in the absence of hypertrophic stimuli (Figure 5C), indicating that the R123W mutation in fact converted CRYAB from a suppressor to maladaptive activator of calcium-dependent signaling. To test whether CRYAB^R123W^ also promotes calcium-dependent signaling *in vivo*, we crossed *Cryab*^R123W^ mice to a NFAT-luciferase reporter line in which transgenic firefly luciferase expression is driven by a myosin heavy chain promoter and NFAT enhancer elements (45). Consistent with published results (45), NFAT-luciferase activity in control hearts from adult mice remained minimally detected at steady state. In contrast, hearts from *Cryab*^R123W/R123W^ mice exhibited enhanced NFAT-luciferase activity (Figure 5D). Of note, hyperactivation of calcium-dependent signaling was only observed in homozygous animals and in cardiomyofibroblast lines overexpressing CRYAB^R123W^, suggesting that the ability of CRYAB^R123W^ to promote calcium signaling may be copy-number dependent. This dose dependency may also account for the weaker manifestation of hemodynamic changes at steady-state seen in *Cryab*^R123W/+^ mice compared to *Cryab*^R123W/R123W^ littermates (Figures 2D,F). Mechanistically, whether CRYAB^R123W^ promotes nuclear localization of NFAT by hyperactivating calcineurin remains to be tested. Nevertheless, these data demonstrated that CRYAB^R123W^ drives pathologic calcium-dependent signaling, which is normally suppressed by wild-type CRYAB in response to pressure overload.

## Discussion

In this study, we generated a novel HCM mouse model carrying the *Cryab*^R123W^ allele identified in monozygotic twins who developed concordant HCM. Mice expressing the CRYAB^R123W^ variant manifest many pathologic features of HCM: early enhanced systolic function that spontaneously degenerates into diastolic dysfunction and marked cardiac fibrosis triggered by maladaptive hypertrophy. Subsequent mechanistic studies unexpectedly uncovered calcineurin as a protein interactor of CRYAB and demonstrated that the R123W variant in fact enhances NFAT signal transduction, which is otherwise physiologically suppressed by wild-type CRYAB in response to pressure overload. Intriguingly, spontaneous HCM phenotypes were not observed in mice carrying the *Cryab*^R123W^ allele, regardless of zygosity. This is in stark contrast to the concordant clinical manifestation of HCM in the pair of monozygotic twins heterozygous for the *CRYAB*^R123W^ allele. As the initial genetic study focused on non-synonymous variants of known HCM-associated genes (30), it is conceivable that other extremely rare variants may principally drive HCM pathogenesis, with *CRYAB*^R123W^ serving a facilitative role. Alternatively, the discrepancy in HCM phenotypes may arise from intrinsic physiologic differences between mouse and human hearts, considering that many mouse genetic models of HCM in fact do not exhibit spontaneous HCM phenotypes.

*Cryab* is expressed across various tissues with highest expression in lens, skeletal and cardiac muscles. While a germ-line *Cryab*^R123W^ knock-in allele best approximates the genetic configuration in HCM patients, whether the CRYAB^R123W^ variant exerts pathogenic effects principally in a cardiomyocyte-intrinsic manner cannot be easily discerned. Recent single cell RNA-sequencing studies unveiled global alteration in cell-cell communication networks in HCM patients (50–52), even in those where pathogenic drivers are considered to be cardiomyocyte-restricted proteins (unpublished data). Thus, future single cell transcriptomic analysis of *Cryab*^R123W^ hearts may allow identification of key cellular players implicated in this new mouse model of HCM. Conditional expression of the CRYAB^R123W^ variant in those cell types may further help elucidate the contribution of non-cardiomyocyte cellular constituents to HCM development.

Prior studies have demonstrated the critical role of CRYAB in suppressing pathologic calcium signaling induced by pressure-overload (14). Our data further suggest that such function may be mediated via protein-protein interaction between CRYAB and Calcineurin. Unexpectedly, this cardioprotective effect is transformed into a pathogenic one with a single amino acid substitution, R123W. How CRYAB and the R123W variant modulate calcineurin activity, if at all, remains unclear. A recent structural study uncovered that both R120 and R123 are located in the β7–8 sheets of the crystallin domain (53). However, whereas R120 maintains solubility of CRYAB via forming salt bridges with D109 of the dimerization partner (53), R123 does not appear to participate in such activity. It is thus perhaps not surprising that the R123W variant did not induce protein aggregation compared to its R120G counterpart. Besides providing dimerization/oligomerization interface with other crystallin-domain-containing proteins (54–56), the crystallin domain also appears to regulate the chaperone activity of the unstructured N- or C-terminal tails. A recent report demonstrated that the R120G mutation in fact alters structural dynamics of the N-terminal domain, thereby prematurely activating its chaperone function (24). Whether R123W mutation also impacts dimerization and/or alters functions of the flexible N- or C-terminal domain remains to be determined.

In addition to modulating the calcineurin-NFAT axis, *CRYAB* has been implicated in a variety of pathologic processes associated with cardiomyopathies, including autophagy (18, 19), apoptosis (20) and redox balance (41, 57). While a large array of proteins have been demonstrated as CRYAB-interactors (58, 59), a complete inventory of CRYAB interaction partners in cardiac tissues has not been done. Notably, an unbiased characterization of the protein interactome of HSBP2, another family member of the crystallin-domain containing proteins, in heart tissues uncovered many previously unknown mitochondrial binding partners involved in ATP generation and redox balance (60). Thus, future profiling of protein interaction clienteles of CRYAB and CRYAB^R123W^, coupled with comparison of proteomics and phosphoproteomics studies between the two genotypes at steady-state and in response to pressure-overload may provide a comprehensive understanding of their functions in HCM pathogenesis.

Lastly, owing to its ability to bind multiple pro-inflammatory serum proteins from patients with various autoimmune diseases (58), the therapeutic potential of CRYAB as an anti-inflammatory agent has been extensively explored. Unexpected, administration of purified CRYAB was sufficient to mitigate inflammation in several mouse models with minimal toxicity (61–63). Conceivably, identification of a minimal calcineurin-interacting domain may thus allow development of novel therapeutic peptide for HCM patients with evidence of increased calcium signaling. This work thus supports the novel concept that precision targeting of intracellular signaling in HCM may be therapeutically important and add to the disease-specific pharmacologic armamentarium beyond myosin inhibitors.

## Methods

### Mice

Myh6/NFAT-luc [FVB-Tg(Myh6/NFAT-luc)1Jmol/J] reporter mice were purchased from Jackson Laboratories. The *Cryab*^R123W^ and *Mybc3*^Trunc^ mice were generated at the Maine Health Institute for Research Mouse Genome Modification Core Facility https://mhir.org/?page_id=233. The *Cryab*^R123W^ allele was generated via homology directed repair using the CRISPR/Cas9 system. Briefly, a guide RNA (GATCCACATCGGCTGGGATCCGG), single-stranded oligodeoxynucleotides (ssODN) donor template containing the CGG to TGG mutation, and mRNA encoding Cas9 were co-injected into the cytoplasm/pronucleus of single cell embryos. Chimeric founders were first screened for Cas9-mediated genomic targeting by PCR using the primer pair: Cryab-F1-GGGGCCTTTCACCACTAGACT and Cryab-R1-TTGAGCACCTTCCGGTATGAG, followed by restriction fragment length polymorphisms analysis using HpaI. PCR fragments from targeted founders were then subject to Sanger sequencing to confirm the desired CGG to TGG mutation. Generation of the *Mybpc3*^Y838X^ allele was attempted by co-injection of a guide RNA (TGCGTAGACTCGCATCTCATAGG), ssODN donor template containing the TAT to TAA mutation, and mRNA encoding Cas9 into cytoplasm/pronucleus of single cell embryos. Founders were screened by PCR using the following primer pair: Mybpc3-F1-GGTAATCCGGGTCTAGATAGCTT and Mybpc3-R2-CAGCCTGAGCTTCTTCGTGTGTA, followed by restriction fragment length polymorphisms analysis using AflII. Subsequent Sanger sequencing of all targeted founders revealed indels rather than the desired substitution, resulting in a translation termination further downstream of the expected Y838 position. The allele carrying a 10 bp deletion is heron designated as *Mybpc3*^Trunc^. Founders with germ-line transmission were maintained on a C57BL/6 background. All mice were handled in accordance with US National Institutes of Health standards, and all procedures were approved by the Tufts University Institutional Animal Care and Use Committee.

### Cell lines

H9c2 cells were purchased from ATCC (CRL-1446) and maintained on Dulbecco's Modified Eagle Medium supplemented with 10% fetal bovine serum. To generate H9c2 cell lines expressing wild-type human CRYAB (hCRYAB^WT^), R120G (hCRYAB^R120G^) or R123W (hCRYAB^R123W^) variants, DNA sequences encoding the variants were inserted to pLenti-puro vector (#39481, Addgene). H9c2 cells were spin-inoculated with 3rd generation empty or hCRYAB-expressing lentivirus, packaged as previously described (64). Transduced cells were selected and maintained on 10 µg/ml puromycin.

### Transverse aortic constriction (TAC) surgery

Pressure overload was produced by constricting the transverse aorta just after the first great vessel as previously described (65). Ten- to twelve-week-old *Cryab*^+/+^, *Cryab*^R123W/+^ and *Cryab*^R123W/R123W^ mice were subject to severe (27G needle) TAC operation and recovered for five weeks.

### *In vivo* left ventricular hemodynamic studies

*In vivo* left ventricular function was assessed by pressure-volume analysis (66). Briefly, 12- to 16-week-old *Cryab*^+/+^, *Cryab*^R123W/+^ and *Cryab*^R123W/R123W^ mice were anesthetized with 2.5% isoflurance. A 1.4-French PV catheter (SPR-839; Millar Instruments) was advanced across the aortic valve into the left ventricle. The absolute volume was calibrated, and pressure-volume data was assessed at steady state and during preload reduction. Hemodynamics were recorded and analyzed with IOX software (EMKA instruments, Falls Church, VA). Investigators were blinded to genotype during performance of hemodynamic studies and analysis of data.

### Echocardiography

Echocardiography and pulse wave velocity were conducted using Doppler ultrasound (Vevo 2100, VisualSonics). Mice were anesthetized with isoflurane and placed in a recumbent position with paws in contact with pad electrodes for ECG recording on a heated platform (37°C) and maintained with ∼2.0% isofluorane during the procedure to maintain heart rate of 450–550 bpm. For cardiac function, left ventricular end-diastolic and -systolic diameter as well as interventricular septum and posterior wall thickness were determined by averaging values from at least 5 cardiac cycles obtained by M-mode with the short axis view (66). Parameters for diastolic function, including E/E′ were derived from transmural inflow Doppler indexes obtained in an apical four-chamber view or the left ventricular long axis view (67). For animals subject to TAC surgery, cardiac functional parameters were assessed before TAC and weekly starting two weeks after TAC for five weeks.

### Histological analysis

Whole hearts were fixed in 10% formalin, embedded in paraffin and sectioned at 5 µm thickness. Sections from mid-papillary regions of the left ventricular tissues were stained with Masson trichrome reagent for evaluation of cardiac fibrosis or hematoxylin and eosin for assessment of cardiomyocyte size as well as left ventricular wall thickness. Brightfield images were acquired using an Olympus BX40 microscope and SPOT Insight camera and software. Subsequent area measurement was performed in an ImageJ software (NIH).

### Immunoprecipitation

Approximately 40 million H9c2 cells were lysed with 2 ml of non-denaturing lysis buffer (20 mM Tris-HCl pH 8.0, 137 mM NaCl, 1% Nonidet P-40, 2 mM EDTA) in the presence of a protease inhibitor cocktail (G6521, Promega) and phenylmethylsulfonyl fluoride (PMSF) at 50 µg/ml at 4°C on a rotator for 20 min, followed by centrifugation at 12,000 rpm for 10 min. A 40 µl sample of clarified lysate was taken as input. Equal amount of lysate was added to four tubes containing 20 µl of Dynabeads protein G (10003D, ThermoFisher Scientific) and lysis buffer was added to a total of 1 ml, followed by incubation with 1 µg of anti-Cryab (ab76467, Abcam) antibody, anti-calcineurin A antibody (2,614, Cell signaling), or rabbit IgG (2,729, Cell Signaling) on a rotator at 4°C for 16 h. After unbound protein was removed, protein G beads were washed three times with wash buffer (10 mM Tris-HCl pH 7.4, 1 mM EDTA, 1 mM EGTA, 150 mM NaCl, 1% Triton X-100) in the presence of protease inhibitor cocktail and PMSF at 4°C. After final wash, the protein G beads were resuspended in 20 µl of 1x Laemmli Sample Buffer and boiled for 5 min. Immunoprecipitated proteins were resolved by SDS-PAGE and analyzed by immunoblotting. The following primary antibodies were used for immunoblot assay: anti-Cryab (ab13496, Abcam) and anti-Calcineurin (55,6350, BD Biosciences). The following secondary antibodies were used: horse radish peroxidase-conjugated goat anti-mouse IgG light chain specific (115-035-174, Jackson Immunoresearch) and horse radish peroxidase-conjugated goat anti-mouse IgG Fc (A16084, ThermoFisher Scientific).

### Immunofluorescence microscopy

H9c2 cells expressing human wild-type CRYAB or CRYAB variants were plated in coverslip containing 24-well plates at a density of 10^4^ cells per well. The following day, culture medium was removed, and cells were fixed with 4% paraformaldehyde (Electron Microscopy Sciences) at room temperature for 10 min, followed by permeabilization with 0.1% Triton X-100 in blocking buffer (1% bovine serum albumin in phosphate buffered saline) for 15 min. The following primary antibodies were used: anti-CRYAB (ab76467, Abcam) and anti-DESMIN (ab32362, Abcam). The following secondary antibodies were used: Alexa Fluor 488-conjugated goat anti-rabbit IgG (A-11008, ThermoFisher Scientific) and Alexa Fluor 594-conjugated goat anti-mouse IgG (A-11005, ThermoFisher Scientific). Cells were stained with Hoechst (H3570, ThermoFisher Scientific) before mounting. Immunofluorescent images were acquired using a Nikon A1R confocal microscope. Subsequent color balancing, overlaying, and area measurements were performed in an ImageJ software (NIH).

### Electron microscopy

Samples were fixed overnight in a mixture of 1.25% formaldehyde, 2.5% glutaraldehyde, and 0.03% picric acid in 0.1 M sodium cacodylate buffer, pH 7.4. The fixed tissues were washed with 0.1M sodium cacodylate buffer and post-fixed with 1% osmium tetroxide/1.5% potassium ferrocyanide (in H2O) for 2 h. Samples were then washed in a maleate buffer and post fixed in 1% uranyl acetate in maleate buffer for 1 h. Tissues were then rinsed in ddH20 and dehydrated through a series of ethanol [50%, 70%, 95%, (2×)100%] for 15 min per solution. Dehydrated tissues were put in propylene oxide for five minutes before they were infiltrated in epon mixed 1:1 with propylene oxide overnight at 4°C. Samples were polymerized in a 60°C oven in epon resin for 48 h. They were then sectioned into 80 nm thin sections and imaged on a JEOL 1200EX Transmission Electron Microscope.

### Luciferase reporter assay

To assess NFAT transcriptional activity *in vitro*, a mixture of 9X NFAT-alpha-MHC-Luc (51,941, Addgene) and pRL-TK (E2241, Promega) plasmids at a ratio of 3:1 was transfected into H9c2 cell lines expressing human wild-type CRYAB or CRYAB variants plated at 2000 cells per well in a 96-well plate (165,305, ThermoFisher Scientific) using Lipofectamine Transfection Reagent (18,324,012, ThermoFisher Scientific) according to the manufacturer's instruction. One day after transfection, firefly and renilla luciferase activities were assessed using the Dual-Glo Luciferase Assay System (E2920, Promega) and a plate reader (PR3100 Microplate reader, Bio-Rad). For evaluation of NFAT transcriptional activity ex vivo, whole hearts from six- to eight-week-old *Cryab*^+/+^ Myh6/NFAT-luc, *Cryab*^R123W/+^ Myh6/NFAT-luc and *Cryab*^R123W/R123W^ Myh6/NFAT-luc were dissected into 1–2 mm pieces and lysed in 1 ml of Luciferase Cell Culture Lysis Reagent (E1531, Promega) using a dounce homogenizer, followed by centrifugation at 12,000 g for 5 min. Equal amount of clarified lysate and luciferase assay buffer (E1500, Promega) were mixed in a 96-well plate and firefly luciferase activity was immediately measured by a plate reader.

### Mitochondrial membrane potential assay

Mitochondrial membrane potential in human CRYAB variants-expressing H9c2 cell lines was assessed using a JC-1 assay kit (ab113850, Abcam) according to the manufacturer's instructions.

### Statistical analysis

All statistical measurements are displayed as mean ± S.D. *P*-values were calculated with a Mann-Whitney test for two-group comparisons, by Kruskal-Wallis test for multiple-group comparisons, or by two-way ANOVA for multi-group comparisons over a time course using Prims 8 software.

## Data Availability

The original contributions presented in the study are included in the article/**Supplementary Material**, further inquiries can be directed to the corresponding author.

## References

[1] Maron BJ. Hypertrophic cardiomyopathy: a systematic review. JAMA. (2002) 287:1308–20. 10.1001/jama.287.10.1308 11886323

[2] Marian AJ Roberts R. The molecular genetic basis for hypertrophic cardiomyopathy. J Mol Cell Cardiol. (2001) 33:655–70. 10.1006/jmcc.2001.1340 11273720 PMC2901497

[3] Marian AJ. Modifier genes for hypertrophic cardiomyopathy. Curr Opin Cardiol. (2002) 17:242–52. 10.1097/00001573-200205000-00006 12015473 PMC2775140

[4] Geisterfer-Lowrance AA Kass S Tanigawa G Vosberg HP Mckenna W Seidman CE A molecular basis for familial hypertrophic cardiomyopathy: a beta cardiac myosin heavy chain gene missense mutation. Cell. (1990) 62:999–1006. 10.1016/0092-8674(90)90274-I 1975517

[5] Watkins H Macrae C Thierfelder L Chou YH Frenneaux M Mckenna W A disease locus for familial hypertrophic cardiomyopathy maps to chromosome 1q3. Nat Genet. (1993) 3:333–7. 10.1038/ng0493-333 7981753

[6] Thierfelder L Watkins H Macrae C Lamas R Mckenna W Vosberg HP Alpha-tropomyosin and cardiac troponin T mutations cause familial hypertrophic cardiomyopathy: a disease of the sarcomere. Cell. (1994) 77:701–12. 10.1016/0092-8674(94)90054-X 8205619

[7] Seidman CE Seidman JG. Identifying sarcomere gene mutations in hypertrophic cardiomyopathy: a personal history. Circ Res. (2011) 108:743–50. 10.1161/CIRCRESAHA.110.223834 21415408 PMC3072749

[8] Watkins H Ashrafian H Redwood C. Inherited cardiomyopathies. N Engl J Med. (2011) 364:1643–56. 10.1056/NEJMra0902923 21524215

[9] Maron BJ Maron MS. Hypertrophic cardiomyopathy. Lancet. (2013) 381:242–55. 10.1016/S0140-6736(12)60397-3 22874472

[10] Maron BJ Maron MS Maron BA Loscalzo J. Moving beyond the sarcomere to explain heterogeneity in hypertrophic cardiomyopathy: JACC review topic of the week. J Am Coll Cardiol. (2019) 73:1978–86. 10.1016/j.jacc.2019.01.061 31000001 PMC6550351

[11] Ingles J Burns C Bagnall RD Lam L Yeates L Sarina T Nonfamilial hypertrophic cardiomyopathy: prevalence, natural history, and clinical implications. Circ Cardiovasc Genet. (2017) 10. 10.1161/CIRCGENETICS.116.001620 28408708

[12] Gerull B Klaassen S Brodehl A. The genetic landscape of cardiomyopathies. Genetic Causes of Cardiac Disease. (2019):45–91. 10.1007/978-3-030-27371-2_2

[13] Vicart P Caron A Guicheney P Li Z Prevost MC Faure A A missense mutation in the alphaB-crystallin chaperone gene causes a desmin-related myopathy. Nat Genet. (1998) 20:92–5. 10.1038/1765 9731540

[14] Kumarapeli AR Su H Huang W Tang M Zheng H Horak KM Alpha B-crystallin suppresses pressure overload cardiac hypertrophy. Circ Res. (2008) 103:1473–82. 10.1161/CIRCRESAHA.108.180117 18974385 PMC2610480

[15] Bennardini F Wrzosek A Chiesi M. Alpha B-crystallin in cardiac tissue. Association with actin and desmin filaments. Circ Res. (1992) 71:288–94. 10.1161/01.RES.71.2.288 1628387

[16] Nicholl ID Quinlan RA. Chaperone activity of alpha-crystallins modulates intermediate filament assembly. EMBO J. (1994) 13:945–53. 10.1002/j.1460-2075.1994.tb06339.x 7906647 PMC394896

[17] Wang K Spector A. alpha-crystallin stabilizes actin filaments and prevents cytochalasin-induced depolymerization in a phosphorylation-dependent manner. Eur J Biochem. (1996) 242:56–66. 10.1111/j.1432-1033.1996.0056r.x 8954153

[18] Tannous P Zhu H Johnstone JL Shelton JM Rajasekaran NS Benjamin IJ Autophagy is an adaptive response in desmin-related cardiomyopathy. Proc Natl Acad Sci U S A. (2008) 105:9745–50. 10.1073/pnas.0706802105 18621691 PMC2474535

[19] Bhuiyan MS Pattison JS Osinska H James J Gulick J Mclendon PM Enhanced autophagy ameliorates cardiac proteinopathy. J Clin Invest. (2013) 123:5284–97. 10.1172/JCI70877 24177425 PMC3859422

[20] Mitra A Basak T Datta K Naskar S Sengupta S Sarkar S. Role of alpha-crystallin B as a regulatory switch in modulating cardiomyocyte apoptosis by mitochondria or endoplasmic reticulum during cardiac hypertrophy and myocardial infarction. Cell Death Dis. (2013) 4:e582. 10.1038/cddis.2013.114 23559016 PMC3641337

[21] Bagneris C Bateman OA Naylor CE Cronin N Boelens WC Keep NH Crystal structures of alpha-crystallin domain dimers of alphaB-crystallin and Hsp20. J Mol Biol. (2009) 392:1242–52. 10.1016/j.jmb.2009.07.069 19646995

[22] Laganowsky A Benesch JL Landau M Ding L Sawaya MR Cascio D Crystal structures of truncated alphaA and alphaB crystallins reveal structural mechanisms of polydispersity important for eye lens function. Protein Sci. (2010) 19:1031–43. 10.1002/pro.380 20440841 PMC2868245

[23] Mchaourab HS Godar JA Stewart PL. Structure and mechanism of protein stability sensors: chaperone activity of small heat shock proteins. Biochemistry. (2009) 48:3828–37. 10.1021/bi900212j 19323523 PMC2785012

[24] Woods CN Ulmer LD Janowska MK Stone NL James EI Guttman M HSPB5 disease-associated mutations have long-range effects on structure and dynamics through networks of quasi-ordered interactions. *bioRxiv.* (2022) 2022.2005.2030.493970.

[25] Sacconi S Feasson L Antoine JC Pecheux C Bernard R Cobo AM A novel CRYAB mutation resulting in multisystemic disease. Neuromuscul Disord. (2012) 22:66–72. 10.1016/j.nmd.2011.07.004 21920752

[26] Van Spaendonck-Zwarts KY Van Hessem L Jongbloed JD De Walle HE Capetanaki Y Van Der Kooi AJ Desmin-related myopathy. Clin Genet. (2011) 80:354–66. 10.1111/j.1399-0004.2010.01512.x 20718792

[27] Wang X Osinska H Klevitsky R Gerdes AM Nieman M Lorenz J Expression of R120G-alphaB-crystallin causes aberrant desmin and alphaB-crystallin aggregation and cardiomyopathy in mice. Circ Res. (2001) 89:84–91. 10.1161/hh1301.092688 11440982

[28] Bova MP Yaron O Huang Q Ding L Haley DA Stewart PL Mutation R120G in alphaB-crystallin, which is linked to a desmin-related myopathy, results in an irregular structure and defective chaperone-like function. Proc Natl Acad Sci U S A. (1999) 96:6137–42. 10.1073/pnas.96.11.6137 10339554 PMC26848

[29] Brodehl A Gaertner-Rommel A Klauke B Grewe SA Schirmer I Peterschroder A The novel alphaB-crystallin (CRYAB) mutation p.D109G causes restrictive cardiomyopathy. Hum Mutat. (2017) 38:947–52. 10.1002/humu.23248 28493373

[30] Maron BJ Rowin EJ Arkun K Rastegar H Larson AM Maron MS Adult monozygotic twins with hypertrophic cardiomyopathy and identical disease expression and clinical course. Am J Cardiol. (2020) 127:135–8. 10.1016/j.amjcard.2020.04.020 32430163

[31] Rakowski H Carasso S. Quantifying diastolic function in hypertrophic cardiomyopathy: the ongoing search for the holy grail. Circulation. (2007) 116:2662–5. 10.1161/CIRCULATIONAHA.107.742395 18056537

[32] Vikstrom KL Factor SM Leinwand LA. Mice expressing mutant myosin heavy chains are a model for familial hypertrophic cardiomyopathy. Mol Med. (1996) 2:556–67. 10.1007/BF03401640 8898372 PMC2230192

[33] Ertz-Berger BR He H Dowell C Factor SM Haim TE Nunez S Changes in the chemical and dynamic properties of cardiac troponin T cause discrete cardiomyopathies in transgenic mice. Proc Natl Acad Sci U S A. (2005) 102:18219–24. 10.1073/pnas.0509181102 16326803 PMC1298915

[34] Lowey S Lesko LM Rovner AS Hodges AR White SL Low RB Functional effects of the hypertrophic cardiomyopathy R403Q mutation are different in an alpha- or beta-myosin heavy chain backbone. J Biol Chem. (2008) 283:20579–89. 10.1074/jbc.M800554200 18480046 PMC2459289

[35] Vakrou S Fukunaga R Foster DB Sorensen L Liu Y Guan Y Allele-specific differences in transcriptome, miRNome, and mitochondrial function in two hypertrophic cardiomyopathy mouse models. JCI Insight. (2018) 3. 10.1172/jci.insight.94493 29563334 PMC5926940

[36] Barefield D Kumar M Gorham J Seidman JG Seidman CE De Tombe PP Haploinsufficiency of MYBPC3 exacerbates the development of hypertrophic cardiomyopathy in heterozygous mice. J Mol Cell Cardiol. (2015) 79:234–43. 10.1016/j.yjmcc.2014.11.018 25463273 PMC4642280

[37] Marstrand P Han L Day SM Olivotto I Ashley EA Michels M Hypertrophic cardiomyopathy with left ventricular systolic dysfunction: insights from the SHaRe registry. Circulation. (2020) 141:1371–83. 10.1161/CIRCULATIONAHA.119.044366 32228044 PMC7182243

[38] Beltrami M Bartolini S Pastore MC Milli M Cameli M. Relationship between measures of left ventricular systolic and diastolic dysfunction and clinical and biomarker status in patients with hypertrophic cardiomyopathy. Arch Cardiovasc Dis. (2022) 115:598–609. 10.1016/j.acvd.2022.07.002 36272967

[39] Sanbe A Osinska H Saffitz JE Glabe CG Kayed R Maloyan A Desmin-related cardiomyopathy in transgenic mice: a cardiac amyloidosis. Proc Natl Acad Sci U S A. (2004) 101:10132–6. 10.1073/pnas.0401900101 15220483 PMC454177

[40] Maloyan A Sanbe A Osinska H Westfall M Robinson D Imahashi K Mitochondrial dysfunction and apoptosis underlie the pathogenic process in alpha-B-crystallin desmin-related cardiomyopathy. Circulation. (2005) 112:3451–61. 10.1161/CIRCULATIONAHA.105.572552 16316967 PMC1398051

[41] Alam S Abdullah CS Aishwarya R Morshed M Nitu SS Miriyala S Dysfunctional mitochondrial dynamic and oxidative phosphorylation precedes cardiac dysfunction in R120G-alphaB-crystallin-induced desmin-related cardiomyopathy. J Am Heart Assoc. (2020) 9:e017195. 10.1161/JAHA.120.017195 33208022 PMC7763772

[42] Chou C Chin MT. Pathogenic mechanisms of hypertrophic cardiomyopathy beyond sarcomere dysfunction. Int J Mol Sci. (2021) 22:8933–46. 10.3390/ijms22168933 34445638 PMC8396307

[43] Helms AS Alvarado FJ Yob J Tang VT Pagani F Russell MW Genotype-dependent and -independent calcium signaling dysregulation in human hypertrophic cardiomyopathy. Circulation. (2016) 134:1738–48. 10.1161/CIRCULATIONAHA.115.020086 27688314 PMC5127749

[44] Coppini R Ferrantini C Mugelli A Poggesi C Cerbai E. Altered Ca(2+) and Na(+) homeostasis in human hypertrophic cardiomyopathy: implications for arrhythmogenesis. Front Physiol. (2018) 9:1391. 10.3389/fphys.2018.01391 30420810 PMC6215954

[45] Wilkins BJ Dai YS Bueno OF Parsons SA Xu J Plank DM Calcineurin/NFAT coupling participates in pathological, but not physiological, cardiac hypertrophy. Circ Res. (2004) 94:110–8. 10.1161/01.RES.0000109415.17511.18 14656927

[46] Golenhofen N Ness W Koob R Htun P Schaper W Drenckhahn D. Ischemia-induced phosphorylation and translocation of stress protein alpha B-crystallin to Z lines of myocardium. Am J Physiol. (1998) 274:H1457–64. 10.1152/ajpheart.1998.274.5.H1457 9612350

[47] Maksimiuk M Sobiborowicz A Tuzimek A Deptala A Czerw A Badowska-Kozakiewicz AM. alphaB-crystallin as a promising target in pathological conditions—a review. Ann Agric Environ Med. (2020) 27:326–34. 10.26444/aaem/111759 32955210

[48] Head MW Hurwitz L Kegel K Goldman JE. AlphaB-crystallin regulates intermediate filament organization in situ. Neuroreport. (2000) 11:361–5. 10.1097/00001756-200002070-00028 10674487

[49] Chis R Sharma P Bousette N Miyake T Wilson A Backx PH alpha-Crystallin B prevents apoptosis after H2O2 exposure in mouse neonatal cardiomyocytes. Am J Physiol Heart Circ Physiol. (2012) 303:H967–78. 10.1152/ajpheart.00040.2012 22904156 PMC3706333

[50] Codden CJ Chin MT. Common and distinctive intercellular communication patterns in human obstructive and nonobstructive hypertrophic cardiomyopathy. Int J Mol Sci. (2022) 23:946–68. 10.3390/ijms23020946 35055131 PMC8780670

[51] Codden CJ Larson A Awata J Perera G Chin MT. Single nucleus RNA-sequencing reveals altered intercellular communication and dendritic cell activation in nonobstructive hypertrophic cardiomyopathy. Cardiol Cardiovasc Med. (2022) 6:398–415. 10.26502/fccm.92920277 36237479 PMC9555339

[52] Larson A Codden CJ Huggins GS Rastegar H Chen FY Maron BJ Altered intercellular communication and extracellular matrix signaling as a potential disease mechanism in human hypertrophic cardiomyopathy. Sci Rep. (2022) 12:5211. 10.1038/s41598-022-08561-x 35338173 PMC8956620

[53] Shatov VM Muranova LK Zamotina MA Sluchanko NN Gusev NB. alpha-crystallin domains of five human small heat shock proteins (sHsps) differ in dimer stabilities and ability to incorporate themselves into oligomers of full-length sHsps. Int J Mol Sci. (2023) 24:1085–102. 10.3390/ijms24021085 36674601 PMC9860685

[54] Baranova EV Weeks SD Beelen S Bukach OV Gusev NB Strelkov SV. Three-dimensional structure of alpha-crystallin domain dimers of human small heat shock proteins HSPB1 and HSPB6. J Mol Biol. (2011) 411:110–22. 10.1016/j.jmb.2011.05.024 21641913

[55] Delbecq SP Jehle S Klevit R. Binding determinants of the small heat shock protein, alphaB-crystallin: recognition of the “IxI” motif. EMBO J. (2012) 31:4587–94. 10.1038/emboj.2012.318 23188086 PMC3545294

[56] Janowska MK Baughman HER Woods CN Klevit RE. Mechanisms of small heat shock proteins. Cold Spring Harb Perspect Biol. (2019) 11. 10.1101/cshperspect.a034025 30833458 PMC6771367

[57] Rajasekaran NS Connell P Christians ES Yan LJ Taylor RP Orosz A Human alpha B-crystallin mutation causes oxido-reductive stress and protein aggregation cardiomyopathy in mice. Cell. (2007) 130:427–39. 10.1016/j.cell.2007.06.044 17693254 PMC2962423

[58] Rothbard JB Kurnellas MP Brownell S Adams CM Su L Axtell RC Therapeutic effects of systemic administration of chaperone alphaB-crystallin associated with binding proinflammatory plasma proteins. J Biol Chem. (2012) 287:9708–21. 10.1074/jbc.M111.337691 22308023 PMC3322989

[59] Xu W Guo Y Huang Z Zhao H Zhou M Huang Y Small heat shock protein CRYAB inhibits intestinal mucosal inflammatory responses and protects barrier integrity through suppressing IKKbeta activity. Mucosal Immunol. (2019) 12:1291–303. 10.1038/s41385-019-0198-5 31481750

[60] Grose JH Langston K Wang X Squires S Mustafi SB Hayes W Characterization of the cardiac overexpression of HSPB2 reveals mitochondrial and myogenic roles supported by a cardiac HspB2 interactome. PLoS One. (2015) 10:e0133994. 10.1371/journal.pone.0133994 26465331 PMC4605610

[61] Arac A Brownell SE Rothbard JB Chen C Ko RM Pereira MP Systemic augmentation of alphaB-crystallin provides therapeutic benefit twelve hours post-stroke onset via immune modulation. Proc Natl Acad Sci U S A. (2011) 108:13287–92. 10.1073/pnas.1107368108 21828004 PMC3156222

[62] Pangratz-Fuehrer S Kaur K Ousman SS Steinman L Liao YJ. Functional rescue of experimental ischemic optic neuropathy with alphaB-crystallin. Eye (Lond). (2011) 25:809–17. 10.1038/eye.2011.42 21475310 PMC3178147

[63] Velotta JB Kimura N Chang SH Chung J Itoh S Rothbard J alphaB-crystallin improves murine cardiac function and attenuates apoptosis in human endothelial cells exposed to ischemia-reperfusion. Ann Thorac Surg. (2011) 91:1907–13. 10.1016/j.athoracsur.2011.02.072 21619989

[64] Dull T Zufferey R Kelly M Mandel RJ Nguyen M Trono D A third-generation lentivirus vector with a conditional packaging system. J Virol. (1998) 72:8463–71. 10.1128/JVI.72.11.8463-8471.1998 9765382 PMC110254

[65] Richards DA Aronovitz MJ Calamaras TD Tam K Martin GL Liu P Distinct phenotypes induced by three degrees of transverse aortic constriction in mice. Sci Rep. (2019) 9:5844. 10.1038/s41598-019-42209-7 30971724 PMC6458135

[66] Blanton RM Takimoto E Lane AM Aronovitz M Piotrowski R Karas RH Protein kinase g ialpha inhibits pressure overload-induced cardiac remodeling and is required for the cardioprotective effect of sildenafil in vivo. J Am Heart Assoc. (2012) 1:e003731. 10.1161/JAHA.112.003731 23316302 PMC3541610

[67] Gao S Ho D Vatner DE Vatner SF. Echocardiography in mice. Curr Protoc Mouse Biol. (2011) 1:71–83. 10.1002/9780470942390.mo100130 21743841 PMC3130310

