# Corrigendum: a novel αB-crystallin R123W variant drives hypertrophic cardiomyopathy by promoting maladaptive calcium-dependent signal transduction

**DOI:** 10.3389/fcvm.2024.1455263

**Published:** 2024-08-16

**Authors:** Chun Chou, Gregory L. Martin, Gayani Perera, Junya Awata, Amy Larson, Robert Blanton, Michael T. Chin

**Affiliations:** ^1^ Department of Medicine, Tufts University School of Medicine, Boston, MA, United States; ^2^ Molecular Cardiology Research Institute, Tufts Medical Center, Boston, MA, United States

**Keywords:** hypertrophic cardiomyopathy, cardiac hypertrophy, cryab, calcineurin, NFAT, transverse aortic constriction, cardiac fibrosis

**A corrigendum on**
A novel αB-crystallin R123W variant drives hypertrophic cardiomyopathy by promoting maladaptive calcium-dependent signal transduction By Chou C, Martin GL, Perera G, Awata J, Larson A, Blanton R, Chin MT (2023). Front. Cardiovasc. Med. 10:1223244. doi: 10.3389/fcvm.2023.1223244

**Text Correction**

In the published article, there was an error. The methods section inadvertently omitted the sentence “The *Cryab*^R123W^ and *Mybc3*^Trunc^ mice were generated at the Maine Health Institute for Research Mouse Genome Modification Core Facility https://mhir.org/?page_id = 233.”

A correction has been made to **Methods**, *Mice*, Paragraph Number 1. This sentence previously stated:

“Myh6/NFAT-luc [FVB-Tg(Myh6/NFAT-luc)1 Jmol/J] reporter mice were purchased from Jackson Laboratories. The *Cryab*^R123W^ allele was generated via homology directed repair using the CRISPR/Cas9 system. Briefly, a guide RNA (GATCCACATCGGCT GGGATCCGG), single-stranded oligodeoxynucleotides (ssODN) donor template containing the CGG to TGG mutation, and mRNA encoding Cas9 were co-injected into the cytoplasm/pronucleus of single cell embryos.”

The corrected section appears below:

“Myh6/NFAT-luc [FVB-Tg(Myh6/NFAT-luc)1 Jmol/J] reporter mice were purchased from Jackson Laboratories. The *Cryab*^R123W^ and *Mybc3*^Trunc^ mice were generated at the Maine Health Institute for Research Mouse Genome Modification Core Facility https://mhir.org/?page_id = 233. The *Cryab*^R123W^ allele was generated via homology directed repair using the CRISPR/Cas9 system. Briefly, a guide RNA (GATCCACATCGGCT GGGATCCGG), single-stranded oligodeoxynucleotides (ssODN) donor template containing the CGG to TGG mutation, and mRNA encoding Cas9 were co-injected into the cytoplasm/pronucleus of single cell embryos.”

The authors apologize for this error and state that this does not change the scientific conclusions of the article in any way. The original article has been updated.

