# Supplementary material for: A novel αB-crystallin R123W variant drives hypertrophic cardiomyopathy by promoting maladaptive calcium-dependent signal transduction
Source: Front Cardiovasc Med. 2023 Jun 26;10:1223244. doi: 10.3389/fcvm.2023.1223244 (PMC10331725; doi:10.3389/fcvm.2023.1223244)
Supplement: Supplementary file 1 [file Datasheet1.pdf]

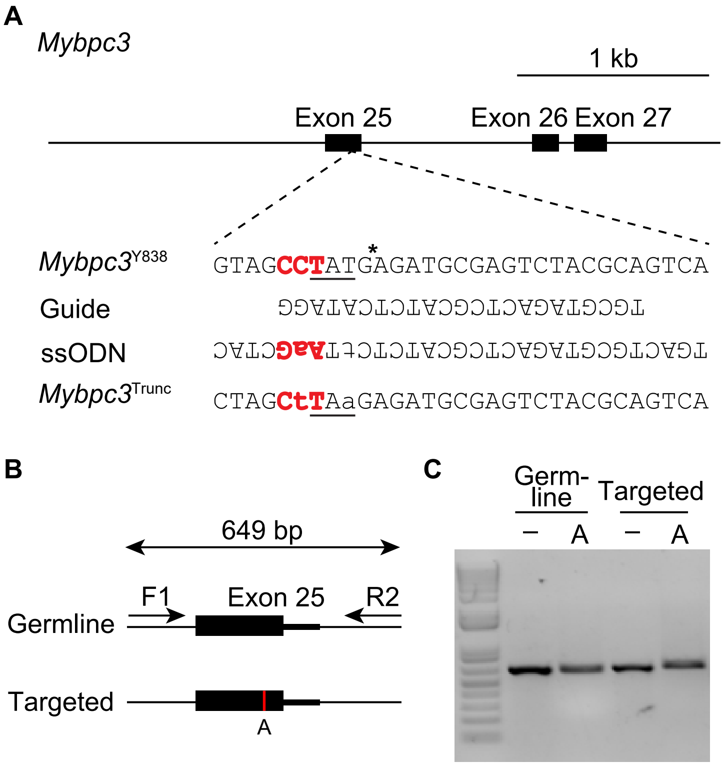

Supplementary Figure 1. Generation of the Mybpc3^{Trunc} allele. (A) A schematic diagram showing strategy to generate a Mybpc3^{Y838X} knock-in allele via CRISPR/Cas9-mediated homology directed repair. The trinucleotides in red denote the protospacer adjacent motif (PAM). The asterisk indicates the cleavage site by the Cas9 nuclease. The underlined trinucleotides encode the target amino acid and the desired nonsense mutation. (B) A screening strategy to identify targeted founders. PCR products of targeted allele differed from germ line configuration by an additional AflII (A) restriction enzyme site, denoted as the red vertical line. (C) Restriction fragment length polymorphisms analysis of germline and targeted animals. PCR fragments spanning the exon of interest before and after AflII digestion were analyzed by agarose gel electrophoresis. PCR fragments from the targeted allele was larger in size than that from germline allele but was not subject to AflII digestion. Subsequent Sanger sequencing of PCR products from the targeted allele revealed the presence of indels rather than the desired nonsense mutation and therefore the allele used in this study, containing a 10bp deletion, is heron designated as Mybpc3^{Trunc}.
